# The Impact of the Pandemic on the Quality of Colorectal and Anal Cancer Care, and 2-Year Clinical Outcomes

**DOI:** 10.3390/curroncol31040173

**Published:** 2024-04-19

**Authors:** Melanie Powis, Rinku Sutradhar, Simron Singh, Shabbir Alibhai, Saidah Hack, Abed Baiad, Kevin Chen, Huaqi Li, Zuhal Mohmand, Monika K. Krzyzanowska

**Affiliations:** 1 Institute for Health Policy, Management and Evaluation, University of Toronto, Toronto, ON M5S 1A1, Canada; rinku.sutradhar@ices.on.ca (R.S.); simron.singh@sunnybrook.ca (S.S.); monika.krzyzanowska@uhn.ca (M.K.K.); 2 Cancer Quality Laboratory (CQuaL), Princess Margaret Cancer Centre, Toronto, ON M5G 1X6, Canada; 3 Institute for Clinical Evaluative Sciences (ICES), Toronto, ON M4N 3M5, Canada; 4 Division of Medical Oncology and Hematology, Sunnybrook Health Sciences Centre, Toronto, ON M4N 3M5, Canada; 5 Department of Medicine, University of Toronto, Toronto, ON M5S 1A1, Canada; kevinjq.chen@mail.utoronto.ca (K.C.); zuhal.mohmand@mail.utoronto.ca (Z.M.); 6 Department of Medicine, University Health Network, Toronto, ON M5G 2C4, Canada; 7 Department of Medicine, McGill University, Montreal, QC H3A 0G4, Canada; abd.baiad@mail.mcgill.ca

**Keywords:** quality of care, COVID-19, pandemic, colorectal cancer, anal cancer, progression

## Abstract

We undertook a retrospective study to compare the quality of care delivered to a cohort of newly diagnosed adults with colon, rectal or anal cancer during the early phase of COVID-19 (02/20–12/20) relative to the same period in the year prior (the comparator cohort), and examine the impact of the pandemic on 2-year disease progression and all-cause mortality. We observed poorer performance on a number of quality measures, such as approximately three times as many patients in the COVID-19 cohort experienced 30-day post-surgical readmission (10.5% vs. 3.6%; SD:0.27). Despite these differences, we observed no statistically significant adjusted associations between COVID-19 and time to either all-cause mortality (HR: 0.88, 95% CI: 0.61–1.27, *p* = 0.50) or disease progression (HR: 1.16, 95% CI: 0.82–1.64, *p* = 0.41). However, there was a substantial reduction in new patient consults during the early phase of COVID-19 (12.2% decrease), which appeared to disproportionally impact patients who traditionally experience sociodemographic disparities in access to care, given that the COVID-19 cohort skewed younger and there were fewer patients from neighborhoods with the highest Housing and Dwelling, ands Age and Labour Force marginalization quintiles. Future work is needed to understand the more downstream effects of COVID-19 related changes on cancer care to inform planning for future disruptions in care.

## 1. Introduction

In response to the Coronavirus Disease 2019 (COVID-19) pandemic, cancer care delivery was modified globally in order to reduce strain on healthcare resources and mitigate the risk of spread [1]. Reported changes varied by jurisdiction and included reducing surgical caseloads, delaying and deferring treatments [2], the use of oral over intravenous therapies and undertaking virtual rather than in-person visits [3,4,5,6,7]. In colorectal cancer, the suspension of routine screening has been linked to delays in diagnosis and treatment, with a greater proportion of patients presenting in the emergency department [8]. In England during the early phase of the pandemic, a 63% reduction in referrals, a 92% reduction in colonoscopies, 3500 fewer colorectal cancer diagnoses and a 31% relative reduction in the number of surgeries relative to the year prior were reported [9].

As many aspects of cancer care cannot be deferred without impacting downstream clinical outcomes, this presents challenges moving forward. Following Hurricane Katrina-related disruptions to cancer care in 2005, 10-year mortality was found to be higher amongst survivors diagnosed with colon cancer within six months of the hurricane than case-matched controls from other jurisdictions during the same time period [10]. While it is still too early to examine the impact of the COVID-19 pandemic on longer-term clinical outcomes, a recent study examining outcomes of a cohort of patients diagnosed in 04–06/2020 found that, after adjusting for age, sex, rural residence and stage, patients diagnosed with colorectal cancer during the early phase of the pandemic had an approximately 20% higher risk of mortality (HR: 1.21; 95% CI: 1.05–1.40) than those diagnosed in 2018 [11]. Understanding the complex inter-play between treatment modifications and their implications for overall clinical outcomes is necessary as we emerge from the pandemic, though the majority of the articles published to date have focused on patterns of cancer care [12], while few have examined impacts on the quality of care, which may result in the worsening of clinical outcomes.

We undertook a retrospective, comparative cohort study to examine the impact of the COVID-19 pandemic on the quality of cancer care delivered to patients newly diagnosed with colon, rectal or anal cancer, as well as clinical outcomes (all-cause death and disease progression). The pandemic may have exacerbated existing disparities in access to timely care and colorectal cancer screening, particularly for patients from racialized groups [13], older adults with cancer and those residing in lower income neighbourhoods [14]. As such, we examined associations between patient-level demographic and clinical characteristics and established an overall quality score between the COVID-19 and comparator cohorts.

## 2. Methods

### 2.1. Study Overview and Ethics

This study was undertaken at the Princess Margaret Cancer Centre (PM), a large, urban comprehensive cancer centre in Toronto, Canada in a single-payer, universal healthcare system. Approval was received from the University Health Network (#20-5428) and University of Toronto (#00041187) Research Ethics Boards; individual consent was waived, as data were retrospective and the re-identification risk was low. This study’s findings were reported in accordance with the STROBE guidelines.

### 2.2. Cohort Identification 

The cohort was identified from the PM Cancer Registry and consisted of all patients ≥18 years of age, whose first PM consultation for newly diagnosed colon, rectal or anal cancer was between 1 February 2019 and 31 December 2019 (comparator cohort) or 1 February 2020 and 31 December 2020 (COVID-19 cohort). Patients were excluded from the study if they had benign disease. Patients who were undergoing treatment for two synchronous cancers (e.g., prostate and colon cancer) were also excluded, since this would have impacted the trajectory of their disease and choice of treatment, which reduces comparability to standard clinical cases.

### 2.3. Data Collection

Data were manually abstracted by a team of five trained medical student abstractors into a secure, encrypted Research Electronic Data Capture database (REDCap) hosted at the University Health Network [15,16]. For quality measure evaluation, data were collected for encounters and treatments in the 6 months following the patient’s first consultation at PM. Disease progression was defined as initiating a new treatment regimen, or having a clinical note or imaging scan report identifying new disease. Data on disease progression and mortality were abstracted for the two years following the date of diagnosis.

### 2.4. Data Linkage

Since limited sociodemographic factors are captured in a patient’s chart, data were linked by full postal code to the Canadian 2016 Census data using the Postal Code Conversion File and Census Analyzer Tool [17] to obtain rural vs. urban residence and neighbourhood gross income quintiles. Additionally, data were linked to the Ontario Marginalization Index to obtain neighbourhood quintiles for each of the four composite measures of marginalization derived from dissemination area-level Census data [18]. Households and Dwellings (previously called Residential Instability) is a composite measure of family and housing instability based on the type and density of housing and family characteristics, such as what proportion of the population is living alone and what proportion of the dwellings are rented vs. owned. Material Resources, previously called Material Deprivation, is a composite measure of poverty, based on income, housing quality, education and family structure, which includes measures such as the proportion of the population without a high school diploma and the unemployment rate. The Age and Labour Force, previously called Dependency, is a composite measure of the proportion of residents without employment income, including those receiving disability benefits and the proportion of residents who are seniors or children. Racialized and Newcomer Populations, formerly called Ethnic Concentration, is a composite measure of the proportion of residents who are recent immigrants, as well as those who self-report belonging to a Racialized Group.

### 2.5. Quality Measures

Quality measures were identified based on a prior scoping literature review highlighting pandemic-related changes to cancer care [1]. These cancer care changes were mapped to 16 established quality measures that are routinely evaluated in colorectal cancer and represent the best practice (Supplementary File S1) [19,20,21,22]. Eight additional, novel “pandemic-specific measures” were operationalized to capture pandemic-related changes that have been reported in the literature [1] that do not necessarily represent preferred or best-practice care, such as the use of oral vs. IV therapies, or the use of laparoscopy rather than open surgical resection.

### 2.6. Statistical Analysis

All analyses were carried out in R (version 4.1.2, R Foundation for Statistical Computing; Vienna, Austria). We compared demographic and clinical characteristics of the two cohorts using standardized differences (SDs) rather than *p*-value since the sample size was small and SDs are not directly dependent on sample size, and this allowed us to compare the size of differences between the two cohorts [23,24,25]. Performance on individual quality measures was calculated for each cohort as the proportion of patients meeting the indicator definition (numerator) out of those who were eligible (denominator), and these were compared using SD. SD > 0.1 was considered indicative of a meaningful difference [26,27].

To examine patient-level differences in the overall quality of care, an overall quality score was generated for each patient as a count of the number of process quality measures for which a patient received measure-concordant care out of those they were eligible for; measures were equally weighted. Univariate Poisson regression models were utilized to examine potential unadjusted associations between patient-level sociodemographic and clinical characteristics and overall quality score by cohort, with the natural logarithm of the number of measures they were eligible for as an offset (range: 5–14 measures); *p*-values ≤ 0.05 were considered statistically significant.

Plots of cumulative incidence for each outcome (x-axis) by to days since diagnosis (y-axis) were generated and stratified into COVID-19 and comparator groups. Incidence was estimated at two years post-diagnosis and compared using the log-rank and Gray’s tests as appropriate, whereby *p* < 0.05 was considered significant. Associations between being in the COVID-19 group (vs. the comparator) and time to death, as well as time to progression, with death as a competing risk, were examined using multivariable Cox Proportional Hazards and Fine-Gray regression models, respectively, and these were adjusted for age, disease stage and primary; *p* < 0.05 was considered significant.

## 3. Results

### 3.1. Cohort Description

Relative to the same period in the year prior, there was a 12.2% reduction in new consults during the early phase of the COVID-19 pandemic (COVID-19: 294 patients, comparator: 335; Figure 1). Relative to the comparator, patients in the COVID-19 cohort were younger (mean age: 66.1 vs. 67.5 years; SD: 0.135), and fewer patients resided in neighbourhoods in the highest marginalization quintile of the Household and Dwellings (41.5% vs. 44.8%; SD: 0.113) and Age and Labour Force (15.6% vs. 23.9%; SD: 0.214) categories, or highest socioeconomic status (21.8% vs. 23.6%; SD: 0.117; Table 1). Additionally, fewer patients were treated with surgery during COVID-19 (41.8% vs. 48.7%; SD: 0.143) compared with the year prior.

### 3.2. Changes to Cancer Care Delivery during COVID-19

A greater proportion in the COVID-19 cohort received treatment with short-course radiotherapy (25 Gy in 5 fractions) for rectal cancer compared to the same period in the year prior (32.6% vs. 11.1%; SD: 0.54; Table 2). Relative to the same period in the year prior, greater proportions of patients in the COVID-19 cohort experienced an interruption in their systemic therapy or radiotherapy (32.4% vs. 22.3%; SD: 0.23) or had their radiotherapy treatment prematurely discontinued (9.4% vs. 1.1%; SD: 0.38). No significant differences were observed in the proportions of patients being treated with oral or single-agent systemic therapy, having the initiation of their treatment deferred or participating in an interventional clinical trial (SD: 0.01–0.09).

### 3.3. Quality Measure Performance

Relative to the year prior, fewer colon cancer patients (82.4% vs. 90.9%; SD: 0.25; Table 2) and anal cancer patients (65.5% vs. 88.0%; SD: 0.55) received the appropriate oncologist consultations. Fewer patients with rectal (24.7% vs. 33.3%; SD: 0.19) and anal cancers (61.5% vs. 85.7%; SD: 0.57) received the appropriate treatment during COVID-19 compared to the year prior. In the 30 days following surgery, there were three times as many patients with all-cause readmission in the COVID-19 cohort compared to the same period in the year prior (10.5% vs. 3.6%; SD: 0.27). For patients whose first consultation was with a medical oncologist or radiation oncologist, a greater proportion in the COVID-19 cohort received a consultation with their oncologist within two weeks of referral than in the comparator cohort (84.9% vs. 72.4%; SD: 0.31). For patients treated with systemic therapy, the proportion who experienced death within 30 days of treatment was significantly lower in the COVID-19 cohort than in the comparator (1.0% vs. 13.8%; SD: 0.51). A greater proportion of patients were diagnosed with cancer in the emergency department during COVID-19 than in the year prior (30.3% vs. 27.8%; SD: 0.19). Additionally, fewer patients in the COVID-19 cohort had a pathology report in their medical record that confirmed disease (68.0% vs. 74.6%; SD: 0.20).

The mean overall quality performance was lower in the COVID-19 cohort than the comparator (mean proportion (standard deviation): 57.1% (18.7) vs. 60.8% (17.8); SD: 0.20). Relative to patients presenting with stage I disease at diagnosis, patients with stage IV disease had approximately 25% lower overall quality scores in both cohorts (COVID-19-IRR: 0.74, 95% CI: 0.64–0.87; comparator- IRR: 0.76, 95% CI: 0.66–0.88; Supplementary File S2).

### 3.4. All-Cause Mortality and Disease Progression

**Figure 2:**
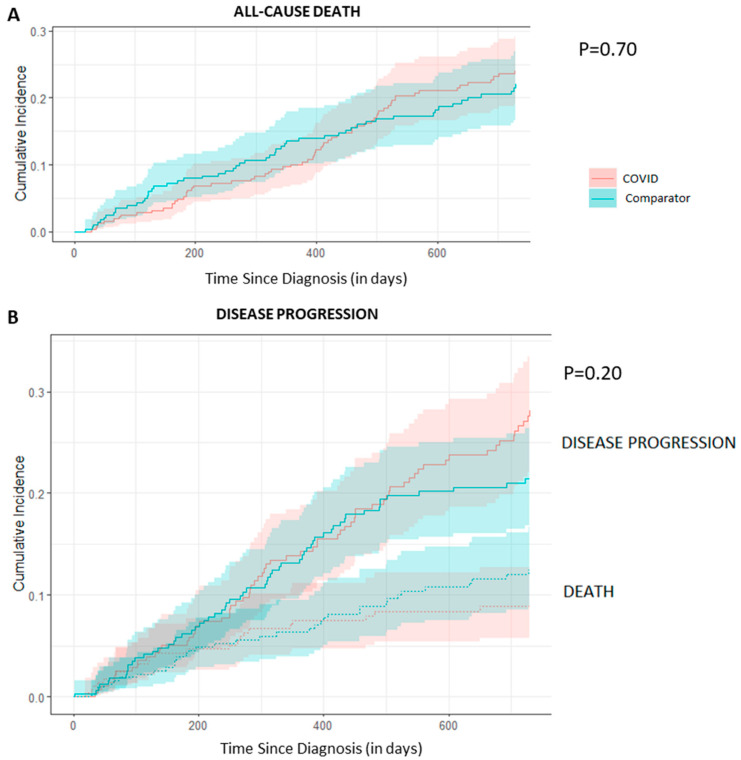
Estimated cumulative incidence of death (**A**) and disease progression with competing risk of death (**B**) by cohort.

Incidence of 2-year mortality (COVID-19: 22.0% [95% CI: 16.6–27], comparator: 24.0% [95% CI: 18.8–28.9]; *p* = 0.70) and disease progression were similar in both groups (COVID-19: 27.1% [95% CI: 22.0–33.0], comparator: 21% [95% CI: 16.2–26.0], *p* = 0.20; Figure 2). When adjusted for age, stage and primary, there was no statistically significant association found between being in the COVID-19 group and time to either all-cause death (HR: 0.88, 95% CI: 0.61–1.27, *p* = 0.50) or disease progression (HR: 1.16, 95% CI: 0.82–1.64, *p* = 0.41; Table 3).

## 4. Discussion

Despite numerous existing quality measures, the literature examining the impact of the pandemic on the quality of cancer care to date has been limited, with the majority of articles focused on the provision of a single modality of treatment, such as surgery or radiotherapy [22,28], or on a specific concept of quality care such as the timeliness of care [29] or patient experience [30]. However, cancer treatment is often multi-modal, and in many jurisdictions, care delivery shifted rather than being altogether cancelled; therefore, a view of the “bigger picture” is needed to truly understand potential downstream implications for patients’ treatment trajectories and prognoses. Relative to the same period in the year prior, we found that there were some differences in quality measure performance for patients treated for colon, rectal or anal cancer during the early phase of COVID-19, though not a profound disruption in care, as was predicted at the onset of the pandemic [31].

Similar to other jurisdictions [32], we found that fewer patients received surgery (41.8% vs. 48.7%; SD: 0.143); however, this decrease was much smaller than in other countries, such as Germany [33], Portugal [34], New Zealand [35] or Australia [36], where 14.5–66% reductions in colorectal surgery have been reported. However, these studies either reported on physician survey data, which may be biased by perceptions surrounding the magnitude of changes in surgical volumes, or their analysis approach was different in that they counted procedures completed relative to the year prior rather than the proportion of patients seen who had had surgery.

We found that there was a greater proportion of patients during COVID-19 who experienced treatment interruption (32.4% vs. 22.3%; SD: 0.23), although this difference was smaller than in other jurisdictions (23.4–70%) [37,38,39]. Similarly, we observed an increase in the proportion of patients treated with short-course radiation for rectal cancer during COVID-19 (32.6% vs. 11.1%; SD: 0.54), which is consistent with the literature [32], although this increase was, again, less pronounced [40]. In contrast with the published literature [1,32], we did not observe differences in the proportions of patients being treated with oral or single-agent therapy, having their treatment start deferred or participating in a clinical trial, although there were few available in this context at the time of study (SD: 0.01–0.09). These findings likely reflect the prioritization of non-elective cancer surgeries in our jurisdiction [41] and lower the overall burden of COVID-19 relative to other countries, resulting in fewer issues with the availability of and access to care [42]. Interestingly, we did observe a decrease in the proportion of patients who died within 30 days of systemic therapy treatment during COVID-19 (1.0% vs. 13.8%; SD: 0.51). This may reflect a change in prescribing behavior to avoid low-value care, may reflect treatments that are less likely to have a clinically meaningful effect [43] or may reflect patient preference.

There are several limitations; this study was carried out at a single, urban cancer centre in a large metropolitan area within a universal healthcare system, which may impact the generalizability of the findings to other jurisdictions and healthcare systems. The comparator was chosen to ensure the feasibility of completion of the manual chart abstraction of the data, to minimize the influence of changes in reorganization of care at the institution or changes in standards of treatment and to allow for appropriate follow-up. The proportion of patients with a pathology report in their medical chart pathologically confirming disease prior to the initiation of treatment was low both during COVID-19 (68.0%) and prior to it (74.6%); however, some records from outside institutions are not uploaded in the chart but, instead, are accessed through a central repository (Connecting Ontario), where access for research purposes is restricted. As such, we may undercount the actual proportion of patients with pathologically confirmed disease. We focused on the quality of cancer care delivered in the six months following the first consultation at PM; therefore, the overlap of care for patients seen as new patient consultations at the end of 2019 and the beginning of the pandemic may have biased our findings towards showing fewer meaningful differences, although very few COVID-19-related changes to care have been reported in patients who initiated treatment prior to the onset of the pandemic [44]. Additionally, we were unable to collect data on patient decision making during COVID-19 as part of this study, which may have influenced the observed differences in patterns of care.

We found a small difference in the mean overall quality score of cancer care delivered during the pandemic relative to the comparator (mean proportion [standard deviation]: 57.1% [18.7] vs. 60.8% [17.8]; SD: 0.20). While COVID-19 has been shown to have compounded existing sociodemographic disparities in cancer care, whereby being of older age [15], from a Racialized Group [13,45,46], or residing in a lower income neighbourhood [14] has been associated with poorer access to screening and treatment, we found no association between patient-level demographic or clinical characteristics and the overall quality score. However, we saw small differences in the sociodemographic characteristics of patients seen during the early phase of COVID-19, whereby they were younger and less likely to be from neighborhoods with the highest Housing and Dwelling, and Age and Labour Force marginalization rates; therefore, patients experiencing the most issues with access to care might have had their care deferred. At 2 years post-diagnosis, we did not observe significant adjusted associations between the pandemic and time to either all-cause mortality (HR: 0.88, 95% CI: 0.61–1.27, *p* = 0.50) or disease progression (HR: 1.16, 95% CI: 0.82–1.64, *p* = 0.41). However, there were relatively few incidences of death and disease progression in our cohorts, resulting in the instability of the estimates. In spite of this, delays in receipt of cancer treatment following the onset of symptoms is associated with poorer prognosis [47]. As such, future work should revisit the impact of the pandemic on the quality of care and clinical outcomes, with a special focus on sociodemographic disparities in the provision of quality care once more time has elapsed.

## 5. Conclusions

While there were some differences in quality measure performance during the early phase of the pandemic, this does not appear to have translated into poorer outcomes after 2 years. However, the reduction in the number of new patients relative to the year prior likely reflects delays and deferrals in the screening and diagnosis of new cancers; therefore, impacts on prognosis may be more latent. As findings following Hurricane Katrina demonstrated that deferred and delayed cancer screening and treatment resulted in an increased 10-year mortality risk [10], our analysis should be repeated once more time (and more events) have elapsed.

## Figures and Tables

**Figure 1:**
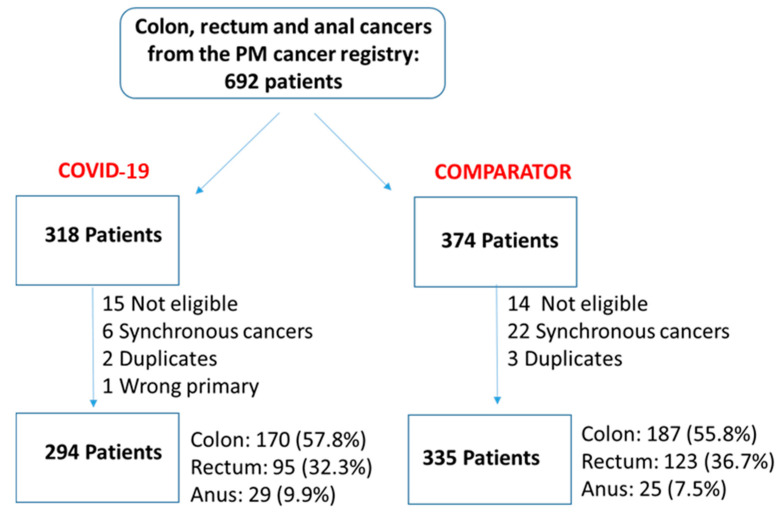
STROBE diagram.

**Table 1:** Summary of demographic and clinical characteristics by cohort.

Variable		COVID-19—2020 (n = 294)	Comparator—2019 (n = 335)	Standardized Difference
**Age**	Mean (SD)	66.1 (15.4)	67.5 (14.9)	**0.14**
**Sex, n (%)**	Male	151 (51.4)	174 (51.9)	0.01
	Female	143 (48.6)	161 (48.1)	
**Marital Status, n (%)**	Married/Common-law Partner	175 (59.5)	205 (61.2)	0.09
	Divorced	14 (4.8)	21 (6.3)	
	Single	49 (16.7)	52 (15.5)	
	Unknown	56 (19.0)	57 (17.0)	
**Language, n (%)**	English as a first language	252 (85.7)	286 (85.4)	0.01
	Other	42 (14.3)	49 (14.6)	
**Highest Level of Education Completed, n (%)**	Less than high school	3 (1.0)	10 (3.0)	**0.18**
	High school	43 (14.6)	39 (11.6)	
	College diploma/Undergraduate degree	24 (8.2)	26 (7.8)	
	Graduate degree	11 (3.7)	14 (4.2)	
	Unknown	213 (72.4)	245 (73.1)	
	Missing	-	1	
**Socioeconomic Status, n (%)**	1—lowest	59 (20.1)	70 (20.9)	**0.12**
	2	54 (18.4)	50 (14.9)	
	3	52 (17.7)	61 (18.2)	
	4	47 (16.0)	58 (17.3)	
	5—highest	64 (21.8)	79 (23.6)	
	Rural	16 (5.4)	14 (4.2)	
	Missing	2 (0.7)	3 (0.9)	
**ON-MARG-Households and Dwellings**	1—least marginalized	44 (15.0)	44 (13.1)	**0.11**
	2	31 (10.5)	42 (12.5)	
	3	37 (12.6)	35 (10.4)	
	4	54 (18.4)	59 (17.6)	
	5—most marginalized	122 (41.5)	150 (44.8)	
	Missing	6 (2.0)	5 (1.5)	
**ON-MARG-Material Resources**	1—least marginalized	81 (27.6)	85 (25.4)	0.08
	2	62 (21.1)	74 (22.1)	
	3	47 (16.0)	62 (18.5)	
	4	53 (18.0)	57 (17.0)	
	5—most marginalized	45 (15.3)	52 (15.5)	
	Missing	6 (2.0)	5 (1.5)	
**ON-MARG- Age and Labour Force**	1—least marginalized	77 (26.2)	83 (24.8)	**0.21**
	2	81 (27.6)	80 (23.9)	
	3	52 (17.7)	57 (17.0)	
	4	32 (10.9)	30 (9.0)	
	5—most marginalized	46 (15.6)	80 (23.9)	
	Missing	6 (2.0)	5 (1.5)	
**ON-MARG- Racialized and Newcomer Populations**	1—least marginalized	26 (8.8)	28 (8.4)	0.08
	2	26 (8.8)	34 (10.1)	
	3	54 (18.4)	62 (18.5)	
	4	84 (28.6)	103 (30.7)	
	5—most marginalized	98 (33.3)	103 (30.7)	
	Missing	6 (2.0)	5 (1.5)	
**Primary, n (%)**	Anal	29 (9.9)	25 (7.5)	**0.11**
	Colon	170 (57.8)	187 (55.8)	
	Rectal	95 (32.3)	123 (36.7)	
**Stage at Diagnosis, n (%)**	I	46 (15.6)	53 (15.8)	**0.16**
	II	50 (17.0)	66 (19.7)	
	III	69 (23.5)	104 (31.0)	
	IV	98 (33.3)	98 (29.3)	
	Unknown	4 (1.4)	11 (3.3)	
	Missing	0 (0)	3 (0.9)	
**ECOG Performance Status, n (%)**	0	47 (16.0)	87 (26.0)	**0.14**
	1	73 (24.8)	113 (33.7)	
	2	12 (4.1)	28 (6.9)	
	3	15 (5.1)	23 (6.9)	
	4	1 (0.3)	1 (0.3)	
	Missing	146 (49.7)	83 (24.8)	
**Treatment, n (%) ^a^**	Surgery	123 (41.8)	163 (48.7)	**0.14**
	Systemic therapy	125 (42.5)	121 (36.1)	0.06
	Radiation therapy	85 (28.9)	87 (26.0)	0.06

^a^ individual patients could count towards multiple categories; ON-MARG: Ontario Marginalization Index; ECOG: Eastern Cooperative Oncology Group.

**Table 2:** Pandemic-specific and quality measure performance.

Measure Type	Measure		COVID-19 Cohort Performance; % (Numerator/Denominator)	Comparator Cohort Performance; % (Numerator/Denominator)	Standardized Difference
**Pandemic-specific Measures**	Receipt of neoadjuvant chemotherapy		16.3 (8/49)	11.8 (8/68)	**0.13**
	Laparoscopic resection		64.6 (62/96)	70.8 (85/120)	**0.13**
	Short-course radiotherapy		32.6 (15/46)	11.1 (6/54)	**0.54**
	Oral systemic therapy		31.2 (39/125)	30.6 (37/121)	0.01
	Single-agent regimens		32.0 (40/125)	30.6 (37/121)	0.03
	Treatment deferral/interruption	Deferral	12.1 (26/214)	9.5 (24/252)	0.09
		Interruption	32.4 (47/145)	22.3 (33/148)	**0.23**
	Premature discontinuation	Systemic therapy	21.6 (27/125)	19.8 (24/121)	0.04
		Radiotherapy	9.4 (8/85)	1.1 (1/87)	**0.38**
	Trial participation		2.7 (8/294)	1.8 (8/335)	0.06
**Quality Measures**	Receipt of appropriate oncology consultation	Colon: surgical oncologist, with or without a consultation with a medical oncologist	82.4 (140/170)	90.9 (170/187)	**0.25**
		Rectum: surgical oncologist AND medical oncologist AND radiation oncologist	22.1 (21/95)	20.3 (25/123)	0.04
		Anus: medical oncologist AND radiation oncologist	65.5 (19/29)	88.0 (22/25)	**0.55**
	30-day post-surgical readmission		10.5 (11/105)	3.6 (5/139)	**0.27**
	Unplanned acute care visit		19.7 (58/294)	23.0 (77/335)	0.08
	Receipt of appropriate treatment	Colon: surgery for stage I–III disease	73.6 (67/91)	77.6 (83/107)	0.09
		Rectum: systemic therapy for stage II–IV disease	24.7 (18/73)	33.3 (31/93)	**0.19**
		Anus: concurrent chemotherapy and radiation for stage I–III disease	61.5 (16/26)	85.7 (18/21)	**0.57**
	Positive margins		3.3 (3/135)	2.2 (4/180)	0.03
	30-day mortality	Systemic therapy	1.0 (1/104)	13.8 (15/109)	**0.51**
		Surgery	1.9 (2/105)	0.7 (1/139)	**0.10**
	Timely oncologist consultation: within 2 weeks of referral		84.9 (73/86)	72.4 (63/87)	**0.31**
	Upstaging		33.7 (98/291)	30.6 (98/320)	0.07
	Timely receipt of treatment: within 60 days of the date of diagnosis		58.2 (85/146)	55.8 (82/147)	0.05
	Diagnosed in emergency department		30.3 (88/290)	27.8 (91/327)	**0.19**
	Pathologically confirmed disease		68.0 (200/294)	74.6 (250/335)	**0.20**
	Receipt of treatment for advanced disease		67.3 (66/98)	70.4 (69/98)	0.07
	Receipt of a palliative care consultation		9.2 (9/98)	9.2 (9/98)	<0.01
	Consent to treatment	Surgery	54.3 (57/105)	58.3 (81/139)	0.08
		Systemic therapy	30.8 (32/104)	27.5 (30/109)	0.07
		Radiotherapy	14.0 (12/86)	14.9 (13/87)	0.03
	Receipt of psychosocial support		14.3 (42/294)	17.9 (60/335)	**0.10**
	Receipt of pain management support		0.0 (0/98)	1.0 (1/98)	**0.14**

**Table 3:** Examining adjusted associations between groups (COVID-19 vs. comparator) and time to all-cause death and disease progression using multivariable Cox proportional hazards and Fine-Gray regression models, respectively.

Variable		Cox Model: All-Cause Death			Fine-Gray Model: Disease Progression					
		HR	95% CI	*p*-Value	Disease Progression			All-Cause Death		
					HR	95% CI	*p*-Value	HR	95% CI	*p*-Value
**Groups**	**Comparator**	Ref	-	-	Ref	-	-	Ref	-	-
	**COVID-19**	0.88	0.61–1.27	0.50	1.16	0.82–1.63	0.41	0.74	0.42–1.28	0.28
**Age**	**5-year increment**	1.22	0.81–1.83	0.30	1.19	0.97–1.45	0.09	1.13	0.95–1.35	0.18
**Primary**	**Colon**	Ref	-	-	Ref	-	-	Ref	-	-
	**Rectal**	0.88	0.58–1.34	0.50	1.14	0.76–1.70	0.52	0.71	0.38–1.35	0.30
	**Anal**	0.79	0.34–1.86	0.60	2.17	1.04–5.40	0.04	0.44	0.10–1.95	0.28
**Stage**	**I**	Ref	-	-	Ref	-	-	Ref	-	-
	**II**	1.03	0.36–2.97	1.00	1.79	0.61–5.24	0.29	1.28	0.31–5.33	0.73
	**III**	2.71	1.13–6.47	0.03	2.57	0.98–6.73	0.06	3.02	0.88–10.4	0.08
	**IV**	7.25	3.11–16.9	<0.01	14.1	5.63–35.40	<0.01	4.38	1.26–15.3	0.02

CI: confidence interval; HR: hazard ratio.

## Data Availability

De-identified data are available upon reasonable, non-commercial request by email to the corresponding author upon execution of a data transfer agreement.

## Supplementary Materials

The following supporting information can be downloaded at https://www.mdpi.com/article/10.3390/curroncol31040173/s1, Supplementary File S1. Quality Measure Definitions. Supplementary File S2. Evaluating potential associations between patient-level sociodemographic and clinical characteristics, and overall quality sore by Poisson regression with score as a count, and the number of measures the patient was eligible for as an offset.
