# Supplementary material for: The Impact of the Pandemic on the Quality of Colorectal and Anal Cancer Care, and 2-Year Clinical Outcomes
Source: Curr Oncol. 2024 Apr 19;31(4):2328–40. doi: 10.3390/curroncol31040173 (PMC11048770; doi:10.3390/curroncol31040173)
Supplement: Supplementary file 1 [file curroncol-31-00173-s001.zip › curroncol-2913036-supplementary.pdf]

Supplementary File 1. Quality Measure Definitions

Type	Measure	Source	Definition	Cohort Restrictions
**Pandemic-Specific Measures**	**Receipt of neoadjuvant chemotherapy**	Scoping literature review[1]	Proportion of patients who received some systemic therapy prior to surgery	Restrict cohort to patients who received surgery for stage III colon or stage II-IV rectal
	**Laparoscopic Resection**	Scoping literature review[1]	Proportion of patients who had surgical resection of colon cancer for which laparoscopy was used	Restrict cohort to only those patients who had a surgical resection for colon<br><br>*Exclude: patients who had biopsy only
	**Short-course radiotherapy**	Scoping literature review[1]	Proportion of patients who received short course radiotherapy for rectal cancer (25Gy, 5 fractions)	Restrict cohort to patients who received radiotherapy for rectal cancer
	**Oral systemic therapy**	Scoping literature review[1]	Proportion of patients on systemic therapy who received at least one oral anti-cancer agent	Restrict cohort to only those patients who received systemic therapy
	**Single Agent Regimens**	Scoping literature review[1]	Proportion of patients who received treatment with a single-agent regimen	Restrict to patients who received systemic therapy
	**Treatment deferral/ interruption**	Scoping literature review[1]	Proportion of patients who experienced a delay or deferral of their treatment<br><br>***Deferral:*** start of a patients' treatment was pushed to a later date as per clinic note or recorded deferral<br><br>***Interruption:*** patients who are on treatment and have an administration held or delayed for any length of time as documented in their medical chart	***Deferral:***<br>restrict to patients receiving treatment (surgery, systemic therapy radiotherapy)<br><br>***Interruption:***<br>Restrict to patients who received at least one dose of systemic therapy or radiotherapy
	**Premature discontinuation**	Scoping literature review[1]	Proportion of patients who stopped treatment prior to completion of their regimen	***Systemic therapy:***<br>Restrict to patients who received systemic therapy<br><br>***Radiotherapy:***<br>Restrict to patients who received radiotherapy
	**Trial Participation**	Scoping literature review[1]	Proportion of patients who participated in an interventional clinical trial	All cohort
**Quality Measures**	**Receipt of appropriate oncology consultation**	ASCO Guidelines	Proportion of patients who received consultation with appropriate oncology services for disease location (radiation, surgical, medical):<br>• ***Colon:*** consultation with a surgical oncologist, with or without a consultation with a medical oncologist	All patients in cohort<br><br>*Stratified by location of disease (colon, rectum, anus)

			Rectum: consultation with each of a surgical oncologist AND medical oncologist AND radiation oncologist ***Anus:*** consultations with each of a medical oncologist AND radiation oncologist *higher proportion= better care	
	**30-day post-surgical readmission**	CSQI	Proportion of patients who had surgical resection that experienced a readmission to our hospital in the 30 days following discharge<br><br>*lower proportion= better care	Restrict cohort to only those patients who had a surgical resection of their primary by a PM surgical oncologist<br><br>*Exclude patients who received a biopsy only
	**Unplanned acute care visit**	CSQI	Proportion of patients who had an unplanned emergency department visit or hospitalization after their first consultation at PM<br><br>*lower proportion= better care	All patients in cohort
	**Receipt of appropriate treatment**	CSQI	proportion of patients who received appropriate treatment<br><br>*3 specific scenarios: - Colon: proportion of patients who received treatment with surgery for stage I-III disease - Rectum: proportion of patients who received treatment with systemic therapy for stage II-IV disease - Anus: proportion of patients who received concurrent chemotherapy and radiation for stage I-III disease *higher proportion= better care	***Colon:*** Patients with stage I-III disease<br><br>***Rectum:*** Patients with stage II-IV disease<br><br>***Anus:*** Patients with stage I-III disease
	**Positive margins**	CSQI	Proportion of patients who have positive surgical margins<br><br>*lower proportion= better care	Restrict to patients who received surgical resection
	**30-day mortality**	ASCO Choosing Wisely	Proportion of patients who die within 30 days of treatment<br><br>*lower proportion= better care	***Systemic Therapy:*** restrict to patients who received at least one dose of systemic therapy<br><br>***Surgery:*** Restrict to patients who received surgical resection at PM<br>*exclude receipt of biopsy only

	**Timely oncologist consultation**	Health Quality Ontario	Proportion of patients that had a consultation with an oncologist within 2 weeks of referral<br><br>*higher proportion= better care	Restrict to patients with a known referral date who's first consult was with medical or radiation oncology
	**Upstaging**	CSQI	Proportion of patients with advanced stage disease at diagnosis<br><br>*lower proportion= better care	All patients in cohort who were staged at diagnosis
	**Timely receipt of treatment**	CSQI	Proportion of patients who initiated treatment within 60 days of the date of their diagnosis<br><br>*higher proportion= better care	Restrict to patients who received systemic therapy or radiotherapy
	**Diagnosed in emergency department**	AHRQ	Proportion of patients who are diagnosed as a result of an emergency department visits or hospitalization<br><br>*lower proportion= better care	Restrict to those patients in the cohort with a known location of diagnosis
	**Pathologically confirmed disease**	ASCO QOPI	Proportion of patients with a pathology report in their medical chart confirming diagnosis<br><br>*higher proportion= better care	All cohort
	**Receipt of treatment for advanced disease**	CPAC	Proportion of patients with stage IV disease who received treatment (surgery, systemic therapy, radiotherapy)<br><br>*higher proportion= better care	Restrict to patients with stage IV disease
	**Receipt of a palliative care consultation**	Canadian Society for Palliative Care Physicians	Proportion of patients with advanced disease who received a consultation with palliative care<br><br>*higher proportion= better care	Restrict to patients with stage IV disease
	**Consent to treatment**	ASCO QOPI	Proportion of patients who have evidence of consent to treatment in their medical record<br><br>*higher proportion= better care	Restrict to those patients who received treatment (surgery, systemic therapy, radiotherapy)<br><br>*stratify by treatment modality
	**Receipt of psychosocial support**	ASCO QOPI	Proportion of patients who have evidence of a psychosocial consultation<br><br>*higher proportion= better care	All cohort
	**Receipt of pain management support**	ASCO QOPI	Proportion of patients who received a consultation for pain management<br><br>*higher proportion= better care	Restrict to patients with stage IV disease

AHRQ: Agency for Healthcare Research and Quality; ASCO: American Society of Clinical Oncology; CPAC: Canadian Partnership Against Cancer; CSQI: Cancer System Quality Index; QOPI: Quality Oncology Practice Initiative

Supplementary File 2. Evaluating potential associations between patient-level sociodemographic and clinical characteristics, and overall quality sore by Poisson regression with score as a count, and the number of measures the patient was eligible for as an offset

Variables		Univariate- COVID			Univariate-Comparator		
		IRR	95% CI	p-value	IRR	95% CI	p-value
**Age**	**5-years**	1.06	0.97-1.18	0.24	1.00	0.98-1.02	0.78
**Sex**	**Male**	Ref	-	-	Ref	-	-
	**Female**	1.05	0.95-1.16	0.35	0.99	0.90-1.08	0.80
**Marital Status**	**Married/ common law**	Ref	-	-	Ref	-	-
	**Divorced**	0.94	0.73-1.20	0.64	1.18	0.98-1.41	0.07
	**Single**	1.07	0.93-1.22	0.34	0.95	0.83-1.08	0.44
	**Unknown**	0.99	0.86-1.13	0.88	0.98	0.86-1.12	0.80
**Language**	**English first language**	Ref	-	-	Ref	-	-
	**Other**	0.97	0.84-1.12	0.70	0.94	0.82-1.07	0.38
**Highest Level of Education Completed**	**Less than high school**	1.10	0.63-1.80	0.73	1.04	0.74-1.44	0.82
	**High school**	0.91	0.70-1.20	0.49	0.86	0.68-1.10	0.22
	**College diploma/ Undergraduate degree**	0.87	0.65-1.18	0.36	0.86	0.67-1.12	0.26
	**Graduate degree**	Ref	-	-	Ref	-	-
	**Unknown**	0.87	0.68-1.12	0.25	0.84	0.68-1.03	0.09
**Socioeconomic Status**	**1- lowest**	0.94	0.81-1.10	0.47	1.02	0.89-1.17	0.76
	**2**	0.97	0.83-1.14	0.75	0.97	0.83-1.12	0.65
	**3**	0.98	0.83-1.14	0.76	0.92	0.80-1.07	0.29
	**4**	1.03	0.88-1.22	0.69	0.98	0.85-1.13	0.81
	**5- highest**	Ref	-	-	Ref	-	-
	**Rural**	1.01	0.79-1.27	0.96	0.91	0.70-1.16	0.44
**ON-MARG: Households and Dwellings**	**1- least marginalized**	Ref	-	-	Ref	-	-
	**2**	0.888	0.72-1.08	0.22	0.97	0.80-1.17	0.74
	**3**	0.82	0.67-1.00	0.06	0.96	0.79-1.16	0.65
	**4**	0.93	0.79-1.11	0.42	0.94	0.79-1.11	0.45
	**5- most marginalized**	0.91	0.79-1.06	0.23	1.03	0.89-1.19	0.70
**ON-MARG: Material Resources**	**1- least marginalized**	Ref	-	-	Ref	-	-
	**2**	0.97	0.84-1.12	0.65	0.99	0.87-1.13	0.92
	**3**	0.89	0.76-1.05	0.18	0.98	0.85-1.12	0.74
	**4**	0.98	0.84-1.14	0.80	1.00	0.86-1.15	0.95
	**5- most marginalized**	0.92	0.78-1.08	0.30	0.98	0.84-1.13	0.75
**ON-MARK: Age and Labour Force**	**1-least marginalized**	Ref	-	-	Ref	-	-
	**2**	0.97	0.84-1.11	0.61	0.98	0.86-1.13	0.82

	**3**	0.96	0.83-1.12	0.63	1.04	0.90-1.20	0.62
	**4**	0.89	0.74-1.07	0.23	1.01	0.84-1.20	0.95
	**5-most marginalized**	0.97	0.83-1.14	0.72	1.09	0.96-1.24	0.20
**ON-MARG: Racialized and Newcomer Populations**	**1- least marginalized**	Ref	-	-	Ref	-	-
	**2**	1.08	0.84-1.38	0.56	1.00	0.80-1.24	0.97
	**3**	1.03	0.84-1.28	0.78	1.00	0.83-1.22	0.99
	**4**	1.07	0.88-1.31	0.53	1.03	0.86-1.24	0.76
	**5- most marginalized**	1.02	0.84-1.25	0.84	1.03	0.86-1.24	0.76
**Primary**	**Colon**	Ref	-	-	Ref	-	-
	**Rectum**	0.91	0.81-1.02	0.12	0.91	0.82-1.00	0.05
	**Anus**	1.13	0.95-1.32	0.15	1.10	0.92-1.31	0.27
**Stage at diagnosis**	**I**	Ref	-	-	Ref	-	-
	**II**	0.98	0.82-1.17	0.83	1.00	0.86-1.18	0.96
	**III**	0.90	0.77-1.06	0.19	1.03	0.89-1.19	0.73
	**IV**	0.74	0.64-0.87	<0.01	0.76	0.66-0.88	<0.01
**ECOG Performance Status**	**0**	Ref	-	-	Ref	-	-
	**1**	0.93	0.80-1.09	0.37	0.99	0.88-1.11	0.88
	**2**	0.68	0.50-0.92	0.01	0.85	0.70-1.02	0.09
	**3**	0.84	0.64-1.09	0.20	0.88	0.72-1.08	0.23
	**4**	1.05	0.41-2.15	0.91	0.97	0.35-2.10	0.95

IRR: incident rate ratio; ON-MARG: Ontario Marginalization Index; ECOG: Eastern Cooperative Oncology Group
